# Comparison of enhanced recovery after surgery protocol and standard protocol for cesarean delivery: A two-center, double-blind, randomized controlled trial

**DOI:** 10.1097/JS9.0000000000005105

**Published:** 2026-03-20

**Authors:** Weijia Du, Hailian Liu, Rui Liu, Chengcheng Xu, Zhendong Xu, Zhiqiang Liu

**Affiliations:** a Department of Anesthesiology, Obstetrics & Gynecology Hospital of Fudan University, Shanghai Key Lab of Reproduction and Development, Shanghai Key Lab of Female Reproductive Endocrine Related Diseases, Shanghai, China; b Department of Anesthesiology, Shanghai First Maternity and Infant Hospital, Tongji University School of Medicine, Shanghai, China

**Keywords:** double-blind RCT, elective cesarean delivery, enhanced recovery after cesarean (ERAC), maternal recovery, spinal anesthesia, standard care, anesthesia

## Abstract

**Background:**

Enhanced recovery after cesarean delivery (ERAC) protocols have been designed to optimize maternal recovery. Although its potential has been demonstrated, high-quality evidence supporting the efficacy of ERAC protocols remains limited. This study aimed to evaluate the effect of anesthesiologist-led bundled ERAC interventions on perioperative adverse events and recovery quality.

**Methods:**

In this two-center, double-blind, randomized controlled trial, 122 women undergoing elective cesarean delivery under spinal anesthesia were allocated to receive either conventional care or a bundled ERAC protocol. The ERAC bundle included: preoperative personalized and information-based education; intraoperative prophylactic phenylephrine infusion, mandatory non-opioid analgesia (intravenous acetaminophen and ketorolac), prophylaxis for postoperative nausea and vomiting (ondansetron/dexamethasone), and active fluid warming; and postoperative scheduled ketorolac. The primary outcome was a composite of perioperative adverse events (hypotension, nausea/vomiting, shivering, pruritus, hypothermia, and moderate-to-severe pain). Secondary outcomes included opioid consumption, length of hospital stay, pain scores at rest and with movement, the ObsQoR-11 recovery score, and anxiety scores.

**Results:**

Among 116 participants who completed the study (57 conventional care, 59 ERAC), the incidence of the primary composite outcome was significantly lower in the ERAC group (45% vs. 71%; absolute risk difference −31.4%, 95% CI −48.1% to −14.7%; odds ratio 0.25, 95% CI 0.11 to 0.56; *P* = 0.001). On postoperative day 1, the ERAC group demonstrated superior recovery, with lower worst pain scores (*P*<0.01), lower pain scores with movement (*P*<0.01), fewer patient-controlled analgesia boluses (*P*<0.01), and higher ObsQoR-11 scores (*P*<0.01). No significant differences were found in pain at rest, length of stay, or anxiety scores.

**Conclusion:**

An anesthesiologist-led, bundled ERAC protocol significantly reduced perioperative adverse events and enhanced the quality of recovery after elective cesarean delivery compared to conventional care.

## Introduction

Enhanced recovery after surgery (ERAS), initially introduced in 1997 for open sigmoid resections, is aimed at reducing the length of hospital stay, decreasing healthcare costs, and improving patient satisfaction^[^1^]^. Since then, the ERAS principles have been widely adopted across multiple surgical specialties. Cesarean delivery (CD), the most common major abdominal surgery worldwide, accounted for approximately 16.3 births per 1000 people in 2023^[^2^]^. In the United States, the CD rate is 32.1% in 2022, with over 1.17 million procedures performed annually^[^3^]^, while China reported an even higher rate of 44.5% as of 2020^[^4^]^. Given the unique challenges faced by postpartum women, including surgical recovery, neonatal care, and breastfeeding, adopting enhanced recovery after cesarean delivery (ERAC) protocols may offer significant benefits by optimizing maternal recovery, promoting mother-infant bonding, and mitigating negative emotional outcomes^[^5–7^]^.

To standardize perioperative care, the Society for Obstetric Anesthesia and Perinatology (SOAP) and the ERAS Society collaborated to develop evidence-based multidisciplinary ERAC guidelines^[^8–11^]^. Despite their increasing adoption, the existing literature on ERAC remains limited, with only three randomized controlled trials (RCTs) evaluating its impact^[^5–12^]^ resulting in insufficient high-quality evidence to draw definitive conclusions. Most ERAC RCTs have focused on traditional clinical endpoints (e.g., length of stay and opioid use). However, ERAC protocols rarely assess outcome measures reported by the patient, such as maternal satisfaction, postpartum quality of recovery, or experience^[^13,14^]^.

The role of anesthesiologists in post-CD recovery has evolved beyond merely ensuring sufficient pain control^[^15^]^. It has progressed from a one-dimensional goal to a more holistic, multidimensional approach to the recovery process, such as the implementation of perioperative guidelines, reflecting a patient-centered perspective on the quality of care. Bundled ERAC interventions associated with obstetric anesthesia have the potential to decrease perioperative adverse events, including hypotension, nausea and vomiting, pain scores, and hypothermia, and ultimately improve the quality of recovery and maternal outcomes. However, robust data supporting the efficacy of ERAC protocols are scarce.

To address this gap, we conducted a two-center, double-blind RCT comparing ERAC with standard care in elective CD with a focus on maternal outcomes. We hypothesized that anesthesiologist-led bundled ERAC interventions would significantly reduce perioperative adverse effects and improve the quality of recovery. This study adopted a novel, patient-centered framework to evaluate the impact of structured perioperative care on maternal well-being.

## Materials and methods

### Study design

This prospective study was approved by the Ethical Committee and followed the Consolidated Standards of Reporting Trials (CONSORT) guidelines^[^16^]^ and the Transparency In The reporting of Artificial INtelligence (TITAN) criteria^[^17^]^. The trial was registered in the Chinese Clinical Trial.

### Study population

For this study, healthy singleton women aged 18–45 years, assigned ASA physical status 1–2, and requesting elective CD under combined spinal-epidural anesthesia were included. All patients had a gestational age of > 37 weeks. The exclusion criteria were as follows: history of pregnancy complications (gestational hypertension, preeclampsia, eclampsia, pregnancy heart disease, gestational diabetes mellitus, pregnancy complicated with liver and kidney diseases, etc.), neuropsychiatric system abnormalities, anxiety and depression, fetal abnormalities, contraindications to neuraxial anesthesia (such as coagulation dysfunction, local anesthetic drug allergy, etc.), chronic pain or opioid abuse; serious adverse events within 24 h after surgery, such as second operation, severe infection, severe intraoperative bleeding (bleeding > 1000 ml), neuraxial anesthesia failure to change to general anesthesia, patients who opted out during the study, and incomplete follow-up data.

### Randomization and concealment of group assignments

Participants were randomly allocated 1:1, using fixed block sizes of four without stratifying factors, to assign them to either the standard protocol (standard group) or enhanced recovery protocol (ERAC group) for CD. An investigator performed a random sequence of group assignments using SAS software (version 9.4; SAS Institute Inc., Cary, NC, USA). Allocation concealment was achieved by staff members not involved in performing the study using numbered, opaque, and sealed envelopes containing group assignments for the participants. After providing verbal and written information and obtaining written consent, eligible participants were assigned a sequential participant identification number that was matched to the number in a randomized envelope.

HIGHLIGHTS

- Cesarean delivery, the most common major abdominal surgery globally, presents distinct challenges to postpartum recovery and maternal health.
- A two-center, double-blind randomized controlled trial was conducted to compare enhanced recovery after cesarean delivery (ERAC) with standard care protocols.
- ERAC protocol significantly reduced perioperative adverse events and enhanced postoperative recovery.

### Blinding

The participants were informed that they were receiving a comprehensive enhanced recovery protocol, but were not informed of the specific group allocation (standard or ERAC) or the details of the intraoperative medication regimen. The attending anesthesiologist administering the spinal injection and the outcome assessor responsible for intraoperative and follow-up data collection were blinded to the group assignments. To maintain blinding, a research nurse who was not involved in intraoperative data collection prepared syringes according to the group allocation.

Standard group:

One 60-mL syringe containing normal saline (total volume: 50 mL)

Two 5-mL syringes containing normal saline (total volume: 2 and 1 mL)

ERAC group:

One 60-mL syringe containing 2 g phenylephrine diluted in normal saline (total volume: 50 mL, 40 µg/mL)

One 5-mL syringe containing 5 mg dexamethasone (total volume: 1 mL)

One 5-mL syringe containing 4 mg ondansetron (total volume: 2 mL)

The intravenous fluid and fluid warming system were concealed under the surgical drapes. The same research nurse connected the fluid to the intravenous line and initiated infusion according to the assigned group, ensuring allocation concealment throughout the procedure.

### Treatment protocol

#### Preoperative management

Enrolled patients participated in a one-to-one educational session with an anesthesia nurse. After obtaining written informed consent, baseline patient information including demographics and medical history was collected. Anxiety levels were assessed using the Self-rating Anxiety Scale (SAS) and Numerical Anxiety Rating Scale (NRS-A). Patients were then randomized to the two study groups to receive the assigned interventions per protocol (detailed in Table 1).

**Table 1:** Perioperative management for both groups.

	Standard group	ERAC group
Preoperative management		
Consultation	Standard pre-anesthetic consultation	Personalized and information-based intervention in addition to standard pre-anesthetic consultation
Fasting	Fasting 6 h for solids and 2 h for fluids	Fasting 6 h for solids and 2 h for fluids
Intraoperative management		
Vasopressor	Bolus of phenylephrine 40 µg or ephedrine 5 mg to treat hypotension	Prophylactic phenylephrine infusion (40 µg/min) + bolus for hypotension
Fluid management	Pre-hydration: 500–1000 mL lactated Ringer’s	Rapid co-hydration: 500–1000 mL lactated Ringer’s
Thermal management	Operating room temperature > 23 °C	Operating room temperature > 23 °C
	Blankets only	Intravenous fluid warming at 40 °C
PONV prophylaxis	At anesthesiologist’s discretion	Mandatory prophylaxis: 4 mg IV ondansetron + 5 mg IV dexamethasone
Intraoperative analgesia	Epidural hydromorphone 0.4–0.6 mg	Epidural hydromorphone 0.4–0.6 mg
		Non-opioid analgesia of 500 mg IV acetaminophen after delivery and 30 mg IV ketorolac after peritoneum closed
Postoperative management		
Multimodal analgesia	Epidural hydromorphone (see above) Acetaminophen 500 mg IV every 6 h, 4 doses Intravenous PCA (oxycodone 40 mg)	Epidural hydromorphone (see above) Ketorolac 30 mg IV every 6 h, 4 doses Acetaminophen 500 mg IV every 6 h, 4 doses Intravenous PCA (oxycodone 40 mg)

ERAC, enhanced recovery after cesarean delivery; IV, intravenous; PCA, patient-controlled analgesia; PONV, postoperative nausea and vomiting.

All participants received the foundational components of our institution’s protocol. The standard care group received routine pre-anesthetic management. The ERAC intervention was applied on top of this foundation and was characterized by a structured, anesthesiologist-led bundle. A key component was enhanced preoperative education, where patients were provided with supplementary materials (see Supplementary Material. http://links.lww.com/JS9/H91) detailing the surgical journey from the operating room to ward recovery and were asked to confirm their comprehension and consent to the plan.

#### Intraoperative management

After transferring the patients to the operating room, non-invasive blood pressure was monitored at 3-minute intervals. Continuous ECG (lead II configuration) and pulse oximetry were monitored throughout the procedure. Body temperature was measured at baseline and immediately post-operation using an infrared tympanic thermometer (Braun IRT6525, accuracy ± 0.2 °F). Intravenous (IV) fluids were warmed to 40 °C using a HOTLINE fluid warmer (Smith Medical, Rockland, USA) as per group allocation.

All patients were administered combined spinal-epidural anesthesia in the left lateral position at the L3-4 interspace, with 12–15 mg of isobaric intrathecal ropivacaine 0.5% (w/v) without opioids. Sensory block height was assessed using ice, and the surgical procedure was initiated once a block above T4 was confirmed. Intravenous carbetocin (100 µg) and epidural hydromorphone (0.4–0.6 mg, diluted to 8 mL with normal saline) were administered in all the patients upon delivery and at the start of skin closure, respectively. The epidural catheter was then removed after the procedure.

#### Postoperative management

Following CD, the patients were permitted to eat and drink at their own discretion upon returning to the ward and were monitored for 24 h. All participants received an intravenous patient-controlled analgesia (PCA) pump (Apon FSQ-IX; Jiangsu Apon Medical Technology, Jiangsu, China) containing oxycodone (0.4 mg/mL). Patients were guided on PCA use in the operating theater and encouraged to utilize it for 24 h. PCA was programmed with a continuous infusion rate of 2 mL/h, supplemented by patient-activated boluses of 4 mL (20-min lockout interval; maximum hourly limit: 10 mL). If patients reported pain ≥4 on a 0–10 numeric rating scale (NRS) after two PCA doses, an additional 500 mg of intravenous acetaminophen was provided.

The indwelling bladder catheter was removed the following morning, after which the patient was mobilized. On the first postoperative day (POD1), pain intensity at rest and during movement was assessed using an NRS scale. Additionally, 24 h after delivery, a research nurse collected data on Obstetric Quality-of-Recovery (ObsQoR-11), anxiety scores of SAS and NRS-A, and data on other side effects such as nausea, vomiting, and pruritus. Patients were also asked to rate their status utilizing a global health NRS, represented as a 100-mm line and ruler, marked at each end with anchors “worst imaginable health state” to “best imaginable health state,” and with “sad” or “happy” stylized representations of faces. Medication administration data were obtained from the electronic medical records on discharge.

### Data collection

Research data were collected using case report forms and recorded in a Microsoft Access database. Baseline blood pressure was calculated as the average of three consecutive measurements taken in the waiting room of the operating theater. Hypertensive episodes, defined as systolic blood pressure exceeding 120% of the baseline value for two consecutive readings, prompted a reduction in the phenylephrine infusion rate. However, the attending anesthesiologist may deviate from this protocol at any time if deemed necessary for patient safety. Patients with spinal anesthesia-induced hypotension (SAIH), defined as a mean arterial pressure (MAP) ≤ 80% of the baseline or a systolic arterial pressure (SAP) < 90 mmHg, were administered a 40-µg intravenous bolus of phenylephrine or ephedrine 5 mg. Severe bradycardia (heart rate <50 beats/min) was managed using intravenous atropine. If nausea and vomiting persisted despite hypotensive treatment, ondansetron (4 mg, IV) was administered. Additionally, patients with hypothermia (body temperature <36.0 °C) received 30 min of preoperative upper-body forced-air warming using an EQ-5000 230 V device (Smiths Medical ASD, Rockland, USA) set to 43 °C.

### Study outcome

#### Primary outcome assessment

The primary outcome of the study was a composite measure including: (1) SAIH; (2) intraoperative or postoperative nausea and vomiting (IONV/PONV); (3) shivering; (4) hypothermia; (5) pruritus and (6) moderate to severe postoperative pain (NRS ≥ 4) within the first 24 h. Each component was assessed as a binary outcome (present/absent), and the occurrence of one or more of these events was considered a positive result for the primary composite outcome.

#### Secondary outcome assessment

Secondary outcomes included delivered/requested PCA bolus, physician rescue analgesic interventions, pain scores (assessed on POD1), length of hospital stay (LOS, defined as time from hospital admission to time of hospital discharge), the ObsQoR-11 score, global health score, and anxiety score, and the incidence of postoperative complications including urinary retention, ileus, wound infection, postpartum hemorrhage, and urinary tract infection.

## Statistical analysis

The sample size was calculated based on the primary outcome. Data from a pilot study including 20 patients suggested that the composite measure of perioperative adverse events was 54.5% in the ERAC group and 77.8% in the control group. A sample size of 54 participants per group provided 80% power to detect this difference, using a two-sided t-test at α = 0.05. To account for 10% of dropouts and catheter failures, 122 participants were enrolled.

Data are presented as median [interquartile range] or number (%), as applicable. Baseline characteristics were compared between the groups using standardized mean differences (SMD). The primary composite outcome was evaluated using chi-square tests, whereas baseline demographics and obstetric characteristics were analyzed using t-tests with SMD. Secondary outcomes, including postoperative pain (highest pain, rest, and motion NRS on POD1), median delivered PCA bolus, quality of recovery (obsQoR-11), global health score, LOS, and postoperative anxiety score, were assessed using Fisher’s exact test (with risk differences) for categorical variables and Mann–Whitney U test (with Hodges-Lehmann shift estimates) for continuous variables. Rescue analgesic use, PONV, shivering, and pruritus were analyzed using chi-square and Mann-Whitney U tests (with Hodges-Lehmann shift) for categorical and continuous measures, respectively.

Group differences were reported as effect estimates with 95% confidence intervals (CIs).

## Results

**Figure 1.**
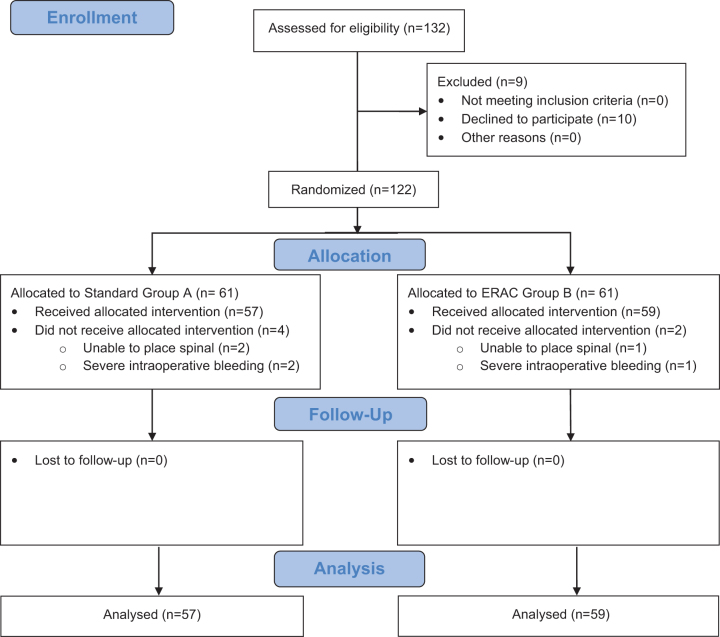
Flow chart showing study participant recruitment.

Among the 132 patients who were assessed for eligibility, 122 were enrolled in the study (61 participants were randomized to each group) (Fig. 1). Six patients were excluded because of neuraxial anesthesia failure (three patients) and intraoperative bleeding of > 1000 mL (three patients). Thus, 57 patients in the standard group and 59 in the ERAC group were included in the primary outcome assessment. Table 2 displays the baseline characteristics of the participants and intraoperative data. There were no clinically important differences between the groups in terms of demographic or obstetric characteristics.

**Table 2:** Baseline demographic and obstetric characteristics.

	ERAC group (n = 59)	Standard group (n = 57)	Standardized mean difference
Age (yr)	34 [31, 36]	33 [30, 35]	0.189
Height (cm)	162 [158, 168]	163 [160, 167]	0.056
Weight (kg)	71 [66, 82]	72 [65, 78]	0.147
Body mass index (kg ∙ m^–2^)	27.0 [25.7, 29.7]	27.3 [24.7, 29.3]	0.137
Gestational age (days)	271 [268, 274]	272 [268, 275]	0.221
Parity	0 [0,1]	0 [0, 0]	0.091
Nulliparous	43 (73)	44 (77)	
Multiparous	16 (27)	13 (23)	
Baseline hemodynamic data			
Systolic blood pressure (mmHg)	121 [112, 126]	121 [114, 130]	0.130
Diastolic blood pressure (mmHg)	74 [68, 79]	73 [67, 81]	0.051
Heart rate (beats/min)	87 [76, 93]	86 [75, 92]	0.140
Temperature (Celsius degree)	36.89 [36.7, 37.0]	36.92 [36.8, 37.1]	0 [−0.1,0]
Baseline anxiety score			
NRS-A	3 [2,4]	3 [2,4]	0.007
SAS	29 [26, 33]	27 [25, 31]	0.240
Intraoperative data and outcomes			
Blood loss (mL)	200 [200, 300]	200 [200, 300]	0.328
Surgery duration (min)	40 [30, 51]	42 [35, 52]	0.164
Rescue oxycodone	1 (2)	2 (4)	0.125
Oxytocin usage	49 (83)	37 (65)	0.417

ERAC, enhanced recovery after cesarean delivery; NRS-A, numerical anxiety rating scale; SAS, self-rating anxiety scale.

Values are mean (SD) or median [interquartile range]. Dichotomous variables were expressed as numbers (percentages).

**Figure 2.**
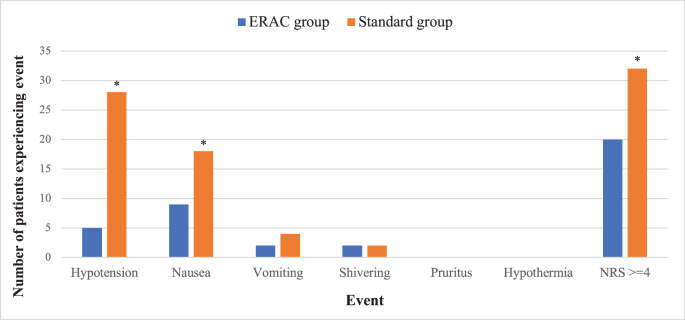
Primary composite outcome and its individual components. *<0.05.

The primary perioperative adverse events demonstrated a statistically significant lower value in the ERAC group than in the standard care group (27/59 [45%] vs. 41/57 [71%]; absolute risk difference [ARD], −31.4% [95% CI, −48.1 to −14.7%]; odds ratio [OR], 0.25 [95% CI, 0.11–0.56]; *P* = 0.001). The primary composite outcome and its individual components are listed in Table 3 and illustrated in Figure 2.

**Table 3:** Primary outcome and its individual components with ERAC protocol vs standard protocol.

	ERAC group (n = 59)	Standard group (n = 57)	Difference measure [95% CI]	Odds ratio [95% CI]	*P* value
Hypotension	5 (9)	28 (49)	0 [−1,0]	0.10 [0.03, 0.28]	<0.001
Nausea	9 (15)	18 (32)	0 [0,0]	0.39 [0.16, 0.96]	0.04
Vomiting	2 (3)	4 (7)	0 [0,0]	0.47 [0.08, 2.64]	0.38
Shivering	2 (3)	2 (2)	0 [0,0]	1.97 [0.17, 2.23]	1.00
Pruritus	0 (0)	0 (0)	0 [0,0]	-	1.00
Hypothermia	0 (0)	0 (0)	0 [0,0]	-	1.00
NRS ≥4	20 (34)	32 (56)	0 [0,0]	2.12[0.19, 23.92]	0.02

CI, confidence intervals; ERAC, enhanced recovery after cesarean delivery; NRS, numeric rating scale.

Dichotomous variables were expressed as numbers (percentages).

The secondary outcomes are presented in Table 4. On POD1, patients in the ERAC group exhibited significantly lower worst pain and motion pain scores compared to those in the standard care group, whereas the rest of the pain scores did not differ significantly between groups. Furthermore, patients in the ERAC group required significantly fewer PCA boluses. No rescue physician analgesic interventions were necessary in either group, and the incidence of adverse effects was comparable between the two groups. The ObsQoR-11 score was significantly higher in the ERAC group, indicating an improved recovery. However, no significant differences were observed in the global health scores, LOS, or 24-hour anxiety scores between the groups.

**Table 4:** Secondary outcomes associated with ERAC protocol vs standard protocol.

	ERAC group (n = 59)	Standard group (n = 57)	Difference measure [95% CI]	*P* value
Pain score in POD1				
Highest pain	3 [2,4]	4 [3,5]	−1 [−1, 0]	0.01
Rest in 24 h	0 [0, 0]	0 [0, 0]	0 [0, 0]	0.17
Motion in 24 h	3 [2,4]	4 [3,5]	−1 [−1, 0]	0.01
Median delivered PCA bolus	2 [1,4]	4 [1,6]	−1 [−3, 0]	0.002
Median requested PCA bolus	2 [1,4]	5 [1,7]	−1 [−3, 0]	0.01
Rescue analgesic intervention	0 (0)	0 (0)	0 [0, 0]	1.000
Side effects in POD1				
Nausea	8 (14)	11 (19)	0 [0, 0]	0.40
Vomiting	3 (5)	8 (14)	0 [0, 0]	0.10
Shivering	0 (0)	2 (4)	0 [0, 0]	0.46
Pruritus	1 (2)	2 (4)	0 [0, 0]	0.98
Quality of recovery				
ObsQoR-11 score	65 [57, 78]	60 [51, 71]	7 [2,13]	0.01
Global health score	80 [80, 90]	80 [75, 85]	0 [0,5]	0.07
LOS (days)	6 [5,7]	6 [5,6]	0 [0,1]	0.35
Anxiety score in POD1				
NRS-A	0 [0, 0]	0 [0, 0]	0 [0, 0]	0.41
SAS	23 [21, 27]	23 [22, 26]	−1 [−2, 0]	0.43

Values are mean (SD) or median [interquartile range]. Dichotomous variables were expressed as numbers (percentages).

CI, confidence intervals; ERAC, enhanced recovery after cesarean delivery; LOS, length of hospital stay; NRS, numeric rating scale; NRS-A, numerical anxiety rating scale; PCA, patient-controlled analgesia; POD1, postoperative day 1; SAS, self-rating anxiety scale.

## Discussion

Our study demonstrates that the anesthesiologist-led bundled ERAC protocol significantly improves the quality of recovery after cesarean delivery compared with standard care, as evidenced by the lower incidence of composite adverse outcomes. The ERAC group also exhibited superior postoperative recovery with significantly reduced worst pain and movement-related pain scores on POD1, fewer PCA bolus requests, and higher ObsQoR-11 scores, indicating better overall recovery.

Recovery after cesarean section is a complex and multifaceted process, with definitions that often differ between clinicians and patients. From a clinical standpoint, the primary focus remains on restoring the essential functions needed to facilitate early hospital discharge. Teigen *et al*^[^5^]^ introduced a comprehensive ERAC protocol that incorporates multiple evidence-based postoperative interventions. Their findings demonstrated that this approach led to a shorter average postoperative hospital stay than standard perioperative care and improved exclusive breastfeeding rates among postpartum women. Similarly, Baluku *et al.*^[^6^]^ established the feasibility of ERAC implementation in emergency cesarean deliveries in resource-limited settings, achieving reduced hospitalization duration without increasing postoperative complication rates. Recently, advancements in recovery assessment tools have shifted from relying solely on one-dimensional physiological measures to incorporating comprehensive continuous evaluations that encompass physical, nociceptive, emotional, cognitive, and functional outcomes^[^18,19^]^. Our results indicate that the implementation of bundled ERAC protocols significantly enhances recovery across multiple domains: physiological (including hemodynamic stability, PONV incidence, and pain scores), psychological (as measured by anxiety assessment scores), and patient-reported functional outcomes (as measured by ObsQoR-11 scores). These findings substantiate the importance of ERAC in achieving a comprehensive postoperative rehabilitation. Through the systematic integration of physiological, psychological, and experiential recovery dimensions, ERAC promotes an optimized, patient-centered recovery trajectory following CD.

Current evidence posits that patient-reported outcome measures (PROMs) are the gold standard for comprehensive postpartum recovery assessment, although their clinical implementation remains suboptimal^[^20^]^. Among the available PROM tools, the ObsQoR score has emerged as the recommended benchmark for evaluating functional recovery after CD. However, despite its validated status and clinical relevance, its utilization in both research and practice settings remains limited^[^21^]^, highlighting a significant gap between evidence-based recommendations and real-world application in postpartum care. A recent RCT found a statistically significant improvement in ObsQoR-11 scores following the implementation of ERAC protocols for elective CD. The median score increased from 82 in the pre-ERAC to 85 in the post-ERAC group^[^22^]^. Another study conducted in India demonstrated mean ObsQoR scores of 84.6 and 75.0 for good and poor recovery, respectively^[^23^]^. In the original study describing its development, the median score was 100 for good recovery and 87 for poor recovery^[^18^]^. However, we observed comparatively lower median scores (60–65 across the standard and ERAC groups), suggesting potential variability in recovery patterns influenced by contextual or cultural factors. One such factor is the traditional Chinese practice of “doing the month” (postpartum confinement)^[^24^]^, which prescribes prolonged rest, restricted ambulation, and avoidance of activities such as infant carrying and bathing due to the perceived risks of cold exposure and subsequent systemic vulnerability. While this practice aligns with the biological imperative of minimizing physiological stress in the early postpartum period, it contrasts with ERAC principles that advocate early mobilization and active neonatal care engagement. Within-cultural differences between Western and Asian countries influence patients’ expectations of recovery. Given the growing emphasis on patient-centered recuperation, further investigation is warranted to examine the feasibility of integrating culturally adapted modifications into evidence-based ERAC frameworks to optimize postpartum rehabilitation across diverse populations. Such consideration of the sociocultural determinants of health would enhance the worldwide generalizability of recovery assessment tools.

Although this study provides valuable insights into the benefits of an ERAC protocol for elective cesarean deliveries, some limitations must be acknowledged. The exclusion of high-risk pregnancies and emergency cesarean deliveries restricts the generalizability of the findings as they may reflect different recovery challenges and complication profiles. Furthermore, the 24-hour postoperative assessment, though useful for capturing early recovery milestones, does not account for long-term outcomes, potentially overlooking delayed complications or the sustainability of recovery benefits. The variability in ObsQoR-11 scores across different populations, particularly when compared to Western studies, highlights the influence of cultural factors on recovery expectations, suggesting that ERAC protocols may require cultural adaptations to optimize patient-centered care in diverse settings. Additionally, while robust blinding was maintained for the anesthesiologists and outcome assessors, complete blinding of participants to a multifaceted intervention involving preoperative education was not feasible. Participants in the ERAC group received a more intensive and structured educational session, which could have influenced their perceptions and expectations. However, the primary outcome was a composite of objective adverse events and standardized pain scores, which were assessed by blinded personnel, thereby strengthening the validity of these findings.

## Conclusion

Implementation of an anesthesiologist-led bundled ERAC protocol significantly enhanced recovery outcomes following CD, as evidenced by reduced adverse events, improved pain control, and higher patient-reported recovery scores. However, to generalize ERAC adoption, future research should focus on investigating ERAC protocols in high-risk and emergency obstetric scenarios, incorporating extended postoperative follow-up to assess long-term recovery, and exploring culturally sensitive modifications to enhance patient adherence and satisfaction. Multicenter studies with standardized ERAC components could improve generalizability, whereas cost-effectiveness analyses would help determine the feasibility in resource-limited settings. Additionally, integrating digital health tools for remote recovery monitoring and patient-reported outcome tracking could further refine the ERAC implementation. By addressing these limitations and expanding research directions, future studies may strengthen the evidence base for ERAC protocols and facilitate their adoption in diverse clinical settings.

## Data Availability

The data that support the findings of this study are not publicly available due to privacy and ethical restrictions. The data are, however, available from the corresponding author, Zhiqiang Liu, upon reasonable request.

## Research registration unique identifying number (UIN)

The trial was registered in the Chinese Clinical Trial Registry (No: ChiCTR2500097236; principal investigator: Weijia Du; date of registration: 14 February 2025).

## Presentation (for original articles only)

Preliminary data (as Comparison of Enhanced Recovery after Surgery Protocol and Standard Protocol for Cesarean Delivery: A Two-center, Double-blind, Randomized Controlled Trial) (Abstract No. 2317641) will be presented as a poster at the Society for Obstetric Anesthesia and Perinatology, 58th annual meeting, April 29 - May 3, 2026, at Le Westin Montreal in Montreal, Quebec, Canada.
